# Trajectories of adherence to mood stabilizers in patients with bipolar disorder

**DOI:** 10.1186/s40345-019-0154-z

**Published:** 2019-09-04

**Authors:** M. Bauer, T. Glenn, M. Alda, R. Bauer, P. Grof, W. Marsh, S. Monteith, R. Munoz, N. Rasgon, K. Sagduyu, P. C. Whybrow

**Affiliations:** 1 Department of Psychiatry and Psychotherapy, Medical Faculty, University Hospital Carl Gustav Carus, Technische Universität Dresden, Fetscherstr. 74, 01307 Dresden, Germany; 2 ChronoRecord Association Inc., Fullerton, CA USA; 3 Department of Psychiatry, Dalhousie University, Halifax, NS Canada; 4 Mood Disorders Center of Ottawa, University of Toronto, Toronto, Canada; 5 Department of Psychiatry, University of Massachusetts, Worcester, MA USA; 6 Michigan State University College of Human Medicine, Traverse City Campus, Traverse City, MI USA; 7 Department of Psychiatry, University of California San Diego, San Diego, CA USA; 8 Department of Psychiatry and Behavioral Sciences, Stanford School of Medicine, Palo Alto, CA USA; 9 Department of Psychiatry, University of Missouri Kansas City School of Medicine, Kansas City, MO USA; 10 Department of Psychiatry and Biobehavioral Sciences, Semel Institute for Neuroscience and Human Behavior University of California Los Angeles (UCLA), Los Angeles, CA USA

## Abstract

**Background:**

Nonadherence with mood stabilizers is a major problem that negatively impacts the course of bipolar disorder. Medication adherence is a complex individual behavior, and adherence rates often change over time. This study asked if distinct classes of adherence trajectories with mood stabilizers over time could be found, and if so, which patient characteristics were associated with the classes.

**Methods:**

This analysis was based on 12 weeks of daily self-reported data from 273 patients with bipolar 1 or II disorder using ChronoRecord computer software. All patients were taking at least one mood stabilizer. The latent class mixed model was used to detect trajectories of adherence based on 12 weekly calculated adherence datapoints per patient.

**Results:**

Two distinct trajectory classes were found: an adherent class (210 patients; 77%) and a less adherent class (63 patients; 23%). The characteristics associated with the less adherent class were: more time not euthymic (p < 0.001) and female gender (p = 0.016). No other demographic associations were found.

**Conclusion:**

In a sample of motivated patients who complete daily mood charting, about one quarter were in the less adherent class. Even patients who actively participate in their care, such as by daily mood charting, may be nonadherent. Demographic characteristics may not be useful in assessing individual adherence. Future research on longitudinal adherence patterns in bipolar disorder is needed.

## Introduction

Mood stabilizers are a fundamental treatment for both acute episodes of bipolar disorder and the prevention of future episodes. Patient nonadherence is an important contributor to medication nonresponse (Osterberg and Blaschke 2005), and nonadherence with mood stabilizers may negatively impact every aspect of bipolar disorder. Poor medication adherence is associated with an increase in relapses (Gutiérrez-Rojas et al. 2010; Franks et al. 2008), suicide and suicide attempts (Gonzalez-Pinto et al. 2006; Pompili et al. 2009), emergency room visits, hospitalizations, involuntary hospitalizations, healthcare costs (Svarstad et al. 2001; Gianfrancesco et al. 2008; Hong et al. 2011; Eaddy et al. 2005; Hassan and Lage 2009; Schuepbach et al. 2008), homelessness (Copeland et al. 2009), and involvement with the criminal justice system (Robertson et al. 2014). The harm from medication nonadherence in bipolar disorder is considerable to both individual patients and society.

Medication adherence refers to the extent to which patients follow the medication regimen prescribed by their physician, often defined as taking ≥ 80% of the prescribed doses (Osterberg and Blaschke 2005). Medication adherence involves three phases: initiation of treatment, implementation or following the dosing regimen, and persistence with treatment (Vrijens et al. 2012; Gellad et al. 2017). Diverse factors and behaviors influence each phase including patient attitudes towards medication, clinical symptoms, adverse reactions, regimen complexity, health literacy, substance misuse, medication costs and forgetfulness (Jawad et al. 2018; García et al. 2016; Levin et al. 2016; Fredericksen et al. 2018). An adherence rate is difficult to measure, and will vary with the definition, methodology, adherence phase, study design and study population (Sajatovic et al. 2010; Levin et al. 2016). Less than half of patients with bipolar disorder are estimated to be fully adherent (García et al. 2016; Levin et al. 2016; Scott and Pope 2002). For most patients with bipolar disorder, adherence is partial or intermittent, and changes over time (Scott and Pope 2002; Jawad et al. 2018). Sometimes patients take all the prescribed doses, sometimes a partial dose, and sometimes none for one or all drugs in the medication regimen.

The most frequent way that psychiatrists evaluate medication adherence in bipolar disorder is to ask the patient (Vieta et al. 2012). However, estimates are often incorrect and optimistic (Stephenson et al. 2012; Baldessarini et al. 2008; Lopez et al. 2017; De las Cuevas et al. 2013). Although physicians frequently adjust medications at visits for bipolar disorder (Hodgkin et al. 2018), it is challenging to differentiate non- or inadequate adherence from nonresponse (Velligan et al. 2009). A lack of recognition of patient nonadherence may lead to higher dosages, medication switches and increasingly complex medication regimens, which may further reduce adherence (Baldessarini et al. 2008; Eaddy et al. 2005; Velligan et al. 2009; Colom et al. 2005).

More information to help assess adherence in patients with bipolar disorder is needed. Adherence studies generally use conventional longitudinal modeling approaches that assume the individuals in a sample come from a homogeneous population, the outcome of interest has a single growth trajectory, and any defined covariates influence each individual in the same way (Jung and Wickrama 2008; Proust-Lima et al. 2017). In contrast, trajectory analysis accommodates a heterogeneous population, and allows the detection of subgroups or latent classes within a sample that have different trajectories of change over time for an outcome of interest (Nagin and Odgers 2010; Lennon et al. 2018; Jung and Wickrama 2008). Trajectory analysis was previously used to assess medication adherence for several chronic conditions (Greenley et al. 2015; de Vries McClintock et al. 2016; Hommel et al. 2017; Blalock et al. 2019). Based on 12 weeks of daily self-reported data from patients with bipolar disorder, the purpose of this study was twofold: (1) to determine whether distinct classes of adherence trajectories could be identified for patients taking mood stabilizers, and (2) if trajectory classes were present, to detect associations with patient characteristics.

## Methods

All data were from outpatients with a diagnosis of bipolar disorder by DSM-IV or DSM-5 criteria that was made by the prescribing psychiatrist during a clinical interview. All participants volunteered, were age 18 years or older, and provided informed written consent. The participants were recruited from a university mood clinic or private practice and received treatment as usual throughout the study. The participants agreed to record medications, mood and sleep daily using computer software in their native language (ChronoRecord). Demographic variables were obtained from the patient by the clinician at the time of enrollment. The demographic characteristics of patients who use ChronoRecord are similar to that for patients in other studies of bipolar disorder (Bauer et al. 2012). Patients were included in this analysis if they had a diagnosis of bipolar I or bipolar II disorder, returned at least 12 weeks of data (84 days), and were taking at least one mood stabilizer during the 12 weeks. Although arbitrary, 12 weeks of data provides sufficient time for adherence analysis, and includes patients that are comfortable with and willing to use ChronoRecord. Data used in this analysis were collected between 2000 and 2016. The ChronoRecord database was previously used for a variety of published analyses. Active collection of ChronoRecord data is ongoing.

### Daily data entry

Patients entered mood, sleep, medications taken and life events daily, and weight weekly into ChronoRecord software (Bauer et al. 2004, 2008). All patients received about a half hour of training in person or by phone before entering data. During training, a medication list was created by selecting from a list of psychotropic medications displayed by brand and generic name. The list includes every medication taken for bipolar disorder. For each selected medication, the pill strength was chosen from a list of available strengths. Every day, for each medication, the patient entered the total number of pills taken. Patients could enter partial pills (1/4, 1/2, or 3/4) for tablets but not capsules. If a medication was not taken, the patient entered zero pills for that drug. The patient could modify the drugs taken throughout the study as needed, and a drug not included in the software list could be added by the patient. Data not entered on one day could be entered later. The software prevents entry for a future date, prevents modification of previously entered data, and requires confirmation for entry of a large number of pills for a drug.

To record mood, patients entered a single daily rating that best described the prior 24 h using a 100-unit visual analog scale. During training, the scale was calibrated to the extremes of mania and depression the patient ever experienced. Based upon the validation studies (Bauer et al. 2004, 2008), a mood entry less than 40 was considered depression, 40–60 euthymia, and greater than 60 hypomania/mania. The range of depression varied between mild symptoms (an entry of 20–39) to moderate to severe symptoms (an entry of 0–19). The range of mania varied from hypomania (an entry of 61–80) to moderate to severe symptoms of mania (an entry of 81–100).

### Drugs analyzed

The mood stabilizers considered were lithium, valproate, lamotrigine, carbamazepine, oxcarbazepine and second generation antipsychotics: aripiprazole, olanzapine, risperidone, quetiapine, ziprasidone, paliperidone, asenapine, lurasidone, and clozapine. For the analysis of total psychotropic drugs taken and the daily pill burden, the other drugs considered were antidepressants, benzodiazepines, typical antipsychotics, insomnia medications, and other anticonvulsants (topiramate, gabapentin, pregabalin, tiagabine, levetiracetam, zonisamide).

### Adherence

All medication data were self-reported, so the prescribed dosage and medication changes were not known. For each patient, adherence was defined as taking at least one pill per day of each prescribed mood stabilizer. An entry of 0 pills or a missing day of data were treated as no pills taken. The adherence was calculated for each day, and then a weekly adherence rate was created for a total of 12 weekly datapoints per patient. For example to calculate a weekly rate, consider a patient taking 2 mood stabilizers. If the patient took at least one pill for drug A on 7 days, and took at least one pill for drug B on 6 days, adherence for that week would be 86% (adherent on 6 days/7 days = 86%). A mood stabilizer that was discontinued by tapering but remained on the medication list was not included in the calculation. If patients returned more than 84 days of data, only the first 12 weeks of data were included to balance the contribution of each patient.

### Latent class trajectory modeling

The latent class mixed model (LCMM), an extension of a standard mixed model, was used to identify the trajectory classes for adherence with mood stabilizers (Proust-Lima et al. 2017; Lennon et al. 2018). Instead of one adherence trajectory for the entire sample, the LCMM allows multiple trajectory classes. LCMM identifies the trajectory classes without predefined assumptions as to the number, size or trajectory pattern of the classes. With LCMM, each patient is included in only one class, and each class has a distinct trajectory based on a supplied trajectory function. The individuals within each trajectory class will be similar to each other, and different from individuals in other classes. The package “lcmm” in R software was used for model estimation (Proust-Lima et al. 2017), and “LCMM toolkit” (Lennon et al. 2018) package was used to verify results.

The analysis models were checked for between 1 and 5 trajectory classes based on a quadratic adherence trajectory function over the 12 weeks. Covariates were not included in the trajectory function, and the variance–covariance matrix was not constrained. The probability that each patient was a member of a class was calculated and all patients were classified according to the highest probability. Several measures were used to help select the optimal number of classes (van de Schoot et al. 2017; Lennon et al. 2018), including the lowest Bayesian Information Criteria (BIC), class size, and probabilities of class membership. The choice of preferred model also included concerns for parsimony and interpretability of results (Jung and Wickrama 2008). To verify the model estimates were not influenced by the initial values, a grid search was performed to confirm the model estimates reflected the best estimates.

For the selected number of classes, the patient characteristics within the classes were compared using Chi-squared tests for categorical variables and ANOVA for continuous variables. Demographic characteristics for the entire sample were also determined. SPSS version 25 was used for all demographic calculations.

## Results

There were 560 patients with bipolar disorder in the database of which 528 had a diagnosis of bipolar I or bipolar II disorder. Of the 528 patients, 483 were taking a mood stabilizer, and 273 returned ≥ 84 days of data and were included in the analysis. The 273 patients included in the analysis resided in the US (189, 69%), Germany (38, 14%), Canada (27, 10%), Australia (7, 3%), Chile (4, 1%), Austria (3, 1%), Poland (3, 1%), and the UK (2, 1%). Of the 273 patients, 151 (55%) were recruited at a university mood center, and 122 (45%) from a private practice. Of the 273 patients, 173 (63.4%) had bipolar I disorder and 100 (36.6%) had bipolar II disorder. Of the 273 patients, 192 (70.3%) were female and 81 (29.7%) were male. The demographic characteristics of 273 patients are shown in Table 1. The 273 patients took a total of 2.8 ± 1.4 psychiatric drugs daily, with a mean pill burden of 6.3 ± 4.3 as shown in Table 2.

**Table 1:** Patient demographics (N = 273)

Category	N	Percent
Gender		
Female	192	70.3
Male	81	29.7
Diagnosis		
BP I	173	63.4
BP II	100	36.6
Disabled		
No	175	73.5
Yes	63	26.5
Working full time		
No	131	55.0
Yes	107	45.0
Any college		
No	32	12.5
Yes	224	87.5
College graduate		
No	115	44.9
Yes	141	55.1
Married		
No	133	52.2
Yes	122	47.8

	Mean	SD
Hospitalizations (N = 248)	2.8	4.51
Age of onset (N = 253)	22.3	10.34
Age (N = 273)	40.8	11.07
Years of illness (N = 253)	18.8	12.12
Percent days depressed (N = 273)	20.5	22.3
Percent days manic/hypomanic (N = 273)	8.4	11.8
Percent days euthymic (N = 273)	64.3	27.5

**Table 2:** Patient medications (N = 273)

Medication	N	Percent
Lamotrigine	117	42.9
Lithium	97	35.5
Valproate	58	21.2
Carbamazepine/oxcarbazepine	34	12.5
Any antipsychotic	123	45.1
Any antidepressant	135	49.5
Any benzodiazepine	58	21.2
Any insomnia medication	18	6.6

	Mean	SD
Total number of medications^a^	2.8	1.4
Total pill burden^a^	6.3	4.3

^a^Only psychiatric drugs

### Latent classes

A comparison of the models identified by LCMM for 1–5 trajectory classes are shown in Table 3. Although the BIC for the 3-class model (− 2108) was slightly smaller than for the 2-class model (− 2091), one of the classes in the 3-class model had only 20 members (7%), the probability of class membership was lower, and trajectories for two of the three classes overlapped, making the interpretation of results more difficult. Therefore the 2-class model was selected as the preferred model.

**Table 3:** LCMM parameter estimates for 1 to 5 classes (N = 273) using a Quadratic Trajectory Function

N classes	Maximum log likelihood	AIC^a^	BIC^b^	Relative entropy	Class parameter	Class 1	Class 2	Class 3	Class 4	Class 5
1	716.85	− 1419.69	− 1394.43		N % APPA^c^	273 100% 1.000				
2^d^	1076.39	− 2130.79	− 2091.09	0.878	N % APPA	210 77% 0.971	63 23% 0.968			
3	1095.99	− 2161.98	− 2107.84	0.889	N % APPA	20 7% 0.965	202 74% 0.968	51 19% 0.905		
4	1095.99	− 2153.98	− 2085.40	0.549	N % APPA	20 7% 0.965	198 73% 0.507	0 0% 0.000	55 20% 0.870	
5	1095.99	− 2145.98	− 2062.97	0.428	N % APPA	20 7% 0.965	0 0% 0.000	0 0% 0.000	193 71% 0.344	60 22% 0.824

Trajectory function was weekly adherence = a_0_ + a_1_ * week + a_2_ * week^2^. No covariates were included

^a^Akaike Information Criterion

^b^Bayesian Information Criterion

^c^Average posterior probability of assignment

^d^Preferred model

**Fig. 1:**
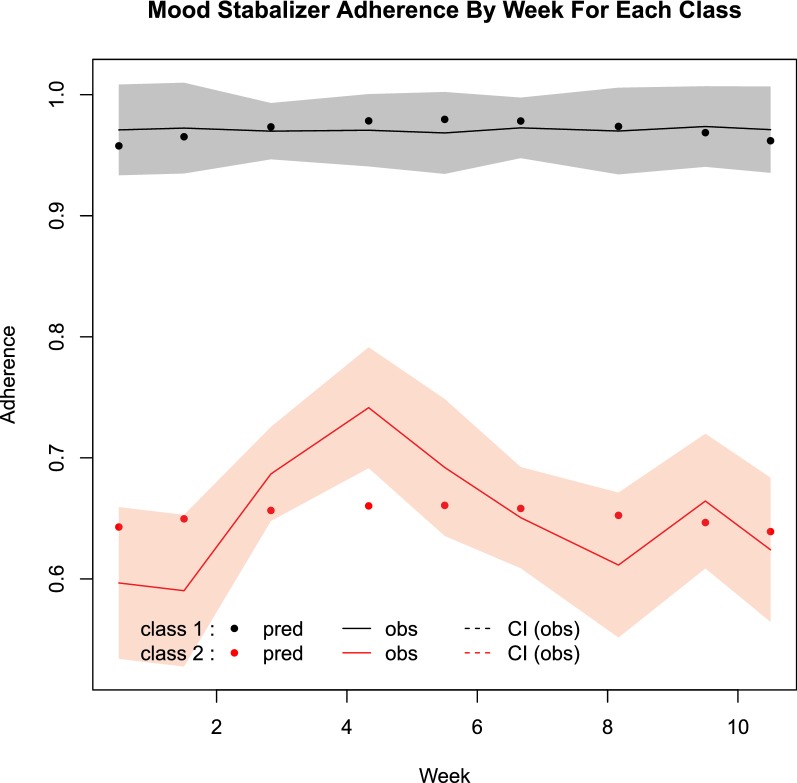
Mood stabilizer adherence by week for each class. *pred* predicted adherence, *obs* observed adherence, *CI* 91% confidence interval

For the preferred model, the actual and predicted weekly adherence trajectory over the 12 weeks for the two classes is shown in Fig. 1. One class has consistently high adherence over the 12 weeks and is referred to as the adherent class, while the other class has consistently lower adherence and is referred to as the less adherent class. The adherent class includes 210 patients or 77% of the group, while the less adherent class includes 63 patients or 23% of the group. The trajectory of the less adherent class was also relatively stable over the 12 weeks.

Only two patient characteristics differed between the classes. The less adherent class spent a smaller percentage of days euthymic, and included more females, as shown in Table 4. There were no significant differences between the two classes in other demographics including age, age of onset, diagnosis, college graduate, marital status, employed full time, number of hospitalizations for bipolar disorder, years of illness, or if receiving government disability payment due to bipolar disorder. There were no significant differences in the total number of psychiatric medications, daily pill burden, specific mood stabilizer taken, or the use of any antidepressant, benzodiazepine, stimulant or insomnia medication. There were no significant differences between the classes in the percentage of days with depression, severe depression, mania/hypomania or mania. The smaller percentage of days euthymic in the less adherent class was due to a larger percentage of days with depressive symptoms in some patients, and to a larger percentage of days with manic/hypomanic symptoms in others.

**Table 4:** Significant differences in the classes by LCMM class (N = 273)

	Class 1 (adherent)		Class 2 (less adherent)		Total		Test	
	N	Percent	N	Percent	N	Percent	Χ^2^ Sig.	DF
Gender								
Male	70	33.3	11	17.5	81	29.7	0.016	1
Female	140	66.7	52	82.5	192	70.3		
Diagnosis								
BP I	135	64.3	38	60.3	173	63.4	0.566	1
BP II	75	35.7	25	39.7	100	36.6		
College graduate								
Yes	108	54.8	33	55.9	141	55.1	0.880	1
No	89	45.2	26	44.1	115	44.9		
Married								
Yes	89	45.9	33	54.1	122	47.8	0.262	1
No	105	54.1	28	45.9	133	52.2		

	Mean	SD	Mean	SD	Mean	SD	F Sig.	DF
Percent days euthymic	68.7	26.0	49.7	27.4	64.3	27.5	< 0.001	1271
Age of onset	22.5	10.3	21.6	10.5	22.3	10.3	0.547	1251
Age	40.9	11.0	40.5	11.5	40.8	11.1	0.834	1271
Total number of medications^a^	2.7	1.4	3.1	1.5	2.8	1.4	0.097	1271
Total pill burden^a^	6.1	4.2	6.9	4.3	6.3	4.3	0.145	1271

^a^Only psychiatric drugs

The mean percent of days missing medication data for all 273 patients was 7.0% ± 13.5. The mean percent of days missing medication data was 2.2% ± 3.4 for the adherent class and 22.9% ± 20.5 for the less adherent class (p < 0.001).

## Discussion

This analysis detected two distinct patterns of adherence with taking mood stabilizers over 12 weeks—an adherent class and a less adherent class. About one quarter of the patients were in the less adherent class. Patients in the less adherent class spent significantly more time with symptoms (not euthymic). In prior research in bipolar disorder, affective morbidity including frequent or severe episodes, psychosis, rapid cycling, and longer standing illness were associated with nonadherence (Leclerc et al. 2013; Greene et al. 2018; Baldessarini et al. 2008; Levin et al. 2016; García et al. 2016). Symptoms that may be most severe during episodes, such as impulsivity (Swann et al. 2008) or lack of insight (Dell’Osso et al. 2002), were also associated with poor adherence (Leclerc et al. 2013, García et al. 2016; Pompili et al. 2009; Belzeaux et al. 2015). Other symptoms such as chaotic lifestyles, loss of daily routine, circadian disruption of sleep–wake cycles, and a preference for nighttime activities may make it harder to adhere with medication regimens (Frank et al. 2000; Melo et al. 2017).

Female gender was also associated with the less adherent class in this study. Although poorer adherence in females with bipolar disorder was reported previously (Kessing et al. 2007; Gianfrancesco et al. 2006; Belzeaux et al. 2013; Murru et al. 2013), review articles show contradictory results for a link between gender and adherence in bipolar disorder: no difference (Colom et al. 2005; Jawad et al. 2018; Greene et al. 2018), inconsistent (Levin et al. 2016), and males more associated with nonadherence (Pompili et al. 2009; Leclerc et al. 2013). In the European Social Survey of 45,700 participants from 24 countries, females were more likely to be nonadherent than males (Stavropoulou 2011). In a study in Japan, females with depression were more likely to hide information related to medication adherence (Sawada et al. 2012). However, the reasons for females being associated with the poor adherence class in this study are unknown.

Other demographic characteristics associated with nonadherence in bipolar disorder in prior research were not significant in this study, including less education, younger age, younger age of onset, and a marital status of single (Leclerc et al. 2013; Levin et al. 2016). Adherence involves a wide range of factors related to the individual, culture, language and communication, disease, drugs, physician, and healthcare system (Leclerc et al. 2013, Pompili et al. 2009; McQuaid and Landier 2018). The routinely collected demographic characteristics may not be associated with adherence, as found in non-psychiatric conditions (Franklin et al. 2016; Steiner et al. 2009) including studies using latent trajectory models (Blalock et al. 2019; de Vries McClintock et al. 2016). Additionally, when significant, the relation between adherence and demographic characteristics is usually weak, providing little practical assistance in discriminating between adherent and nonadherent patients (Steiner et al. 2009; Osterberg and Blaschke 2005). The difficulty in inferring individual adherence based on demographics suggests that self-reported measures may be helpful in clinical settings, and a variety of paper and automated tools are available (Steiner 2012; Stirratt et al. 2015). Review articles report moderate to high correlation between self-reported adherence questionnaires and diaries and electronic monitoring (Monnette et al. 2018; Garber et al. 2004; Shi et al. 2010). While all approaches to measure adherence have strengths and weaknesses (Sajatovic et al. 2010; Lehmann et al. 2014; Levin et al. 2015; Di Matteo 2004), good agreement was found between self-reported measures and serum levels of various psychiatric drugs (Jónsdóttir et al. 2010), and with antidepressants (Loayza et al. 2012).

Given the wide range of behaviors that may impact the initiation, implementation and persistence with treatment, understanding the specific reasons for nonadherence is needed to determine the appropriate remedy (Stirratt et al. 2018; Gellad et al. 2017). The detection of two distinct classes in the current study suggests that future research using trajectory analysis, designed to evaluate nonadherence and define membership in trajectory classes, may increase understanding of patterns of adherence in bipolar disorder.

## Limitations

This analysis overestimates adherence with mood stabilizers for several reasons. By requiring 12 weeks of data, the sample analyzed was pre-selected for adherence. Since taking one pill of a mood stabilizer was considered adherent, patients taking a lower dose than prescribed were included as adherent. The dosage timing and drug administration instructions were also not considered during the calculation. Drugs taken for general medical conditions and over the counter drugs were not included in the analysis. Complex medication regimens and requirements for dosing more than once daily are associated with decreased adherence in a wide range of chronic illness (Ingersoll and Cohen 2008; Coleman et al. 2012). In our prior research, a larger number of psychiatric medications and greater pill burden were associated with irregularity in daily dosage of mood stabilizers and single day omissions (Pilhatsch et al. 2018; Bauer et al. 2013a), but not with adherence when defined as at least one pill of a mood stabilizer a day (Bauer et al. 2010). The 12 week time period would not provide information about long-term persistence with treatment.

There are other limitations to this study. All data were self-reported and there was no objective confirmation of the medication data. There were more females than males. Only oral drugs were included. The optimal rates to define adherence are not uniform, but vary with the pharmacokinetic and pharmacodynamic properties of the drug (Morrison et al. 2017). Adherence to specific mood stabilizers was not investigated, as drug regimens for bipolar disorder are highly customized in clinical practice (Bauer et al. 2013b). Adherence rates may be larger than one if patients take more than the prescribed dose, as noted in some newly admitted inpatients (Geretsegger et al. 2019). The least adherent patients who would not use mood charting, and those who never fill the initial prescription for a mood stabilizer were not included. In a year-long study of 195,930 new electronic prescriptions, the rate of non-filling for adults was 30.2% overall, with 27.7% for drugs classified as neuropsychiatric, and 29.5% for antidepressants (Fischer et al. 2010). ChronoRecord was not designed to evaluate the reasons for nonadherence. Many important variables were not available for analysis relating to patient attitudes, cultural issues, financial concerns, and the use of adherence tools such as pill boxes. Finally, it was not known if patients were receiving any type of psychosocial interventions for bipolar disorder.

## Conclusion

In conclusion, in a sample of motivated patients who complete daily mood charting, there were two trajectories of adherence with taking at least one pill of each prescribed mood stabilizer, adherent and less adherent. About one quarter of the patients were in the less adherent class. Characteristics associated with being in the less adherent class were more time with symptoms (not euthymic), and female gender. Motivated patients may be nonadherent, and demographic characteristics may not be useful to assess individual adherence. Future research to identify longitudinal adherence trajectory patterns is needed in bipolar disorder.

## Data Availability

The data will not be shared or made publicly available. Informed consent for this was not sought from the study participants prior to the collection of data.
